# Pain Management in Pediatric Burns: A Review of the Science behind It

**DOI:** 10.1155/2023/9950870

**Published:** 2023-09-15

**Authors:** Bogdan Ciornei, Vlad Laurentiu David, Diana Popescu, Eugen Sorin Boia

**Affiliations:** ^1^Department of Paediatric Surgery and Orthopedics, “Victor Babes” University of Medicine and Pharmacy, Timisoara, Romania; ^2^Department of Pediatric Surgery, “Louis Turcanu” Emergency Children's Hospital, Timisoara, Romania

## Abstract

Pediatric burns are a significant medical issue that can have long-term effects on various aspects of a child's health and well-being. Pain management in pediatric burns is a crucial aspect of treatment to ensure the comfort and well-being of young patients. The causes and risk factors for pediatric burns vary depending on various factors, such as geographical location, socioeconomic status, and cultural practices. Assessing pain in pediatric patients, especially during burn injury treatment, poses several challenges. These challenges stem from various factors, including the age and developmental stage of the child, the nature of burn injuries, and the limitations of pain assessment tools. In pediatric pain management, various pain assessment tools and scales are used to evaluate and measure pain in children. These tools are designed to account for the unique challenges of assessing pain in pediatric patients, including their age, developmental stage, and ability to communicate effectively. Pain can have significant physical, emotional, and psychological consequences for pediatric patients. It can interfere with their ability to engage in daily activities, disrupt sleep patterns, and negatively affect their mood and behavior. Untreated pain can also lead to increased stress, anxiety, and fear, which can further exacerbate the pain experience. Acute pain, which is short-term and typically associated with injury or illness, can disrupt a child's ability to engage in physical activities and impede their overall recovery process. On the other hand, chronic pain, which persists for an extended period, can have long-lasting effects on physical functioning and quality of life in children. The psychological consequences of burns can persist long after the physical wounds have healed, leading to ongoing emotional distress and impaired functioning. Multimodal pain management, which involves the use of multiple interventions or medications targeting different aspects of the pain pathway, has gained recognition as an effective approach for managing pain in both children and adults. However, it is important to consider the specific needs and considerations of pediatric patients when developing evidence-based guidelines for multimodal pain management in this population. Over the years, there have been significant advances in pediatric pain research and technology, leading to a better understanding of pain mechanisms and the development of innovative approaches to assess and treat pain in children. Overall, pain management in pediatric burns requires a multidisciplinary approach that combines pharmacologic and nonpharmacologic interventions.

## 1. Introduction

Pediatric burns are a significant medical issue that can have long-term effects on various aspects of a child's health and well-being. Pain management in pediatric burns is a crucial aspect of treatment to ensure the comfort and well-being of young patients. The management of pain in pediatric burn patients poses unique challenges due to the severity of the injuries and the need for specialized care.

Burns are excruciatingly painful and necessitate extensive medical attention. This intricacy is influenced by the different severity, etiology, and locations of the injuries, which require a variety of pain control adjustments to offer optimal care. While there are clinical guidelines and procedures in place for pain management in burn patients, the bulk of treatments rely on opioid analgesics as their primary therapeutic intervention. Despite adult research highlighting the potential for developing long-term opioid dependence following burn injury, this population is receiving and being discharged with approximately double the dose of opioids than ten years ago [1].

Over 145,000 occurrences of burn injuries in children under the age of 16 are among the 486,000 burn injuries treated in the United States each year [2].

Effective pain control has been shown in the adult literature to improve patient outcomes. However, reports of equivalent effects in pediatric patients have been scarce, and youngsters have lower rates of analgesia. With opioids remaining at the center of therapy, there has been a drive in recent years to provide a complete, multidisciplinary psychological and physiological approach to pediatric burn treatment. With such a strong emphasis on opioid administration in children's pain treatment, there is a dearth of research examining pediatric burn patient-level features in relation to their use.

This article's scope is to review the available evidence-based approaches to treating pain during burn treatment strategies in children.

## 2. Causes and Risk Factors for Pediatric Burns

Pediatric burns are a significant global health issue, with a higher burden of mortality and morbidity in low- and middle-income countries than in high-income countries [3]. The causes and risk factors for pediatric burns vary depending on various factors, such as geographical location, socioeconomic status, and cultural practices. Understanding these causes and risk factors is crucial for developing effective prevention strategies and improving the care and outcomes of pediatric burn patients. One of the major risk factors for pediatric burns is socioeconomic status. Studies have shown that children from low-income families are at a higher risk of burn injuries due to factors such as overcrowded living conditions, a lack of access to safe housing and infrastructure, and limited parental supervision [4, 5].

In low- and middle-income countries, where the burden of pediatric burns is particularly high, the lack of resources and infrastructure for burn prevention and care further exacerbates the risk [3]. Another significant risk factor for pediatric burns is parental education level. Research has found that children of mothers with low levels of education are at a higher risk of unintentional burns [4]. This may be due to a lack of knowledge and awareness about burn prevention and safety measures.

Education programs targeting parents and caregivers can play a crucial role in reducing the incidence of pediatric burns. The age of the child is also an important risk factor. Young children, particularly those under the age of five, are more vulnerable to burn injuries due to their limited understanding of danger and their natural curiosity. Scald burns from hot liquids, such as boiling water or hot beverages, are the most common type of burn injury in this age group. Older children and adolescents are more likely to sustain burns from flame sources, such as fires and fireworks [3]. The home environment is a significant setting for pediatric burn injuries. Accidents involving fire and contact with hot surfaces are common causes of burns in children. Factors such as open flames, unattended stoves, faulty electrical wiring, and a lack of safety measures contribute to the risk of burn injuries in the home [6].

The COVID-19 pandemic and associated lockdowns have also been found to increase the frequency of burn injuries in children, possibly due to increased time spent at home and changes in daily routines [7]. In addition to these external factors, certain intrinsic factors can also increase the risk of pediatric burns. For example, children with certain medical conditions, such as epilepsy or developmental disabilities, may be more prone to accidents and injuries, including burns [8]. Furthermore, studies have shown that children who have experienced a burn injury are at a higher risk of developing psychopathology, such as post-traumatic stress symptoms or post-traumatic stress disorder (PTSD) [9].

## 3. Classification of Burns

Pediatric burn injuries can be classified based on various factors, including the cause of the burn, the extent and depth of the burn, and the location of the burn on the body. Understanding the classification of pediatric burn injuries is essential for appropriate management and treatment. One way to classify pediatric burn injuries is based on the cause of the burn. Scald burns, caused by hot liquids or steam, are the most common type of burn injury in young children. Flame burns, which result from direct contact with fire or flames, are more common in older children and adolescents [3, 10]. Other causes of pediatric burns include contact burns (direct contact with hot objects or surfaces), electrical burns (resulting from contact with electrical sources), and chemical burns (caused by exposure to corrosive substances) [11].

Another classification of pediatric burn injuries is based on the extent and depth of the burn. The extent of the burn is typically measured as the percentage of total body surface area (TBSA) affected by the burn. Burns can be classified as minor (affecting less than 10% TBSA), moderate (10–20% TBSA), or major (greater than 20% TBSA) [11]. The depth of the burn refers to the layers of skin affected. Superficial burns, also known as first-degree burns, only affect the outermost layer of the skin and typically heal without scarring. Partial-thickness burns, also known as second-degree burns, involve the outer (superficial partial thickness) and inner (deep partial thickness) layers of the skin and may result in blistering and scarring. Full-thickness burns, also known as third-degree burns, extend through all layers of the skin and may require surgical intervention for healing [12]. The location of the burn on the body is another factor used in the classification of pediatric burn injuries. Burns can occur on various parts of the body, including the face, hands, arms, legs, and trunk. The location of the burn can impact the functional and cosmetic outcomes of the injury. For example, burns on the hands or face may result in functional impairments and require specialized care and rehabilitation [9]. In addition, the severity and prognosis of pediatric burn injuries can be classified based on factors such as the presence of an inhalation injury, comorbidities, and the risk of infection. Inhalation injuries, which occur when a child inhales hot gases or smoke, can lead to respiratory complications and significantly impact the prognosis. Comorbidities, such as preexisting medical conditions or injuries, can increase the risk of complications and mortality in pediatric burn patients [13].

The risk of infection is also an important consideration in the classification of pediatric burn injuries, as burn wounds are susceptible to bacterial colonization and subsequent infections. Factors such as burn depth, burn size, and the presence of indwelling devices can increase the risk of infection [14]. In conclusion, pediatric burn injuries can be classified based on the cause of the burn, the extent and depth of the burn, the location of the burn on the body, and factors related to severity and prognosis. Understanding the classification of pediatric burn injuries is crucial for appropriate management, treatment, and prevention strategies.

## 4. Challenges in Assessing Pain in Pediatric Patients

Assessing pain in pediatric patients, especially during burn injury treatment, poses several challenges. These challenges stem from various factors, including the age and developmental stage of the child, the nature of burn injuries, and the limitations of pain assessment tools. One of the challenges in assessing pain in pediatric patients with burn injuries is the difficulty in accurately determining the depth of the burn. Pediatric skin is thinner and has reduced subcutaneous fat stores than that of adult skin, making it more challenging to assess the depth of the burn injury. The thickness of the skin in children is approximately 70% that of adult skin, which complicates the initial assessment of burn depth [15, 16].

An accurate assessment of burn depth is crucial for determining the appropriate treatment and pain management strategies. Another challenge is the subjective nature of pain assessment in pediatric patients, particularly in young children who may not be able to effectively communicate their pain experiences. Verbal pain scales, such as the visual analog scale (VAS), may not be suitable for very young children who have limited language skills [17]. In such cases, alternative pain assessment tools, such as the face pain recognition scale (FPRS) or the face, legs, activity, cry, and consolability (FLACC) scale, may be used to assess pain in nonverbal or preverbal children [18]. Pain assessment in pediatric burn patients is further complicated by the presence of comorbidities such as anxiety and post-traumatic stress disorder (PTSD).

Burn injuries can be traumatic experiences for children, leading to anxiety, fear, and distress. The psychological impact of burn injuries can influence a child's perception and expression of pain, making it challenging to accurately assess their pain levels [9]. In addition, the presence of anxiety and PTSD can exacerbate pain experiences and complicate pain management strategies [19]. The use of sedation and analgesia in pediatric burn patients also presents challenges. Burn injuries can alter the metabolic clearance of sedatives and analgesics, requiring careful consideration of drug dosages and potential drug interactions. The rapid development of tolerance to commonly used sedative agents further complicates pain management in pediatric burn patients. Appropriate sedation and analgesia are crucial for managing the pain, anxiety, and fear associated with burn injuries and their treatment [20]. Furthermore, procedural pain during burn injury treatment poses specific challenges. Dressing changes, wound debridement, and other procedures can cause significant pain and distress in pediatric burn patients. Effective pain management during these procedures is essential to minimize pain and prevent long-term psychological consequences [21]. However, finding the optimal balance between pain control and potential side effects of analgesic medications can be challenging, especially considering the altered drug metabolism in burn patients.

## 5. Common Pain Assessment Tools and Scales

In pediatric pain management, various pain assessment tools and scales are used to evaluate and measure pain in children. These tools are designed to account for the unique challenges of assessing pain in pediatric patients, including their age, developmental stage, and ability to communicate effectively. Some commonly used pain assessment tools and scales in pediatric pain management include the following points.

### 5.1. Adolescent Pediatric Pain Tool (APPT)

The APPT is a multidimensional pain assessment tool specifically designed for adolescents. It assesses pain intensity, location, quality, and impact on daily activities [22, 23].

### 5.2. Pediatric Pain Assessment Tool (PPAT)

The PPAT is a self-report pain tool that assesses pain intensity, location, and quality in children. It is suitable for children aged from 4 to 12 years [22].

### 5.3. Pediatric Pain Questionnaire (PPQ)

The PPQ is a self-report pain tool that assesses pain intensity, location, quality, and impact on daily activities in children aged from 8 to 18 years [22].

### 5.4. Face, Legs, Activity, Cry, and Consolability (FLACC) Scale

The FLACC scale is an observational pain assessment tool that evaluates pain based on facial expressions, leg movements, activity level, cry, and consolability. It is commonly used for nonverbal or preverbal children [24–26].

### 5.5. Wong–Baker FACES Pain Rating Scale

The Wong–Baker FACES scale is a visual analog scale that uses a series of faces to represent different levels of pain intensity. It is a widely used tool for assessing pain in children, including those with limited language skills [27, 28].

### 5.6. Numerical Rating Scale (NRS)

The NRS is a self-reported pain scale that asks children to rate their pain intensity on a numerical scale, typically ranging from 0 to 10 [24].

These pain assessment tools and scales provide healthcare professionals with standardized methods to evaluate and quantify pain in pediatric patients. They help in determining appropriate pain management strategies and monitoring the effectiveness of interventions. It is important to select the most appropriate tool based on the child's age, developmental stage, and ability to communicate effectively. In addition, healthcare professionals should consider cultural and contextual factors that may influence the child's pain experience and expression [24, 28, 29]. It is worth noting that the choice of pain assessment tool may vary depending on the specific clinical setting, the healthcare provider's preference, and available resources. The selection of an appropriate pain assessment tool should be based on evidence-based practice and the individual needs of the pediatric patient.

## 6. Importance of Early and Ongoing Pain Assessment

The importance of early and ongoing pain assessment in pediatric patients cannot be overstated. Pain assessment is a critical component of providing optimal care for children, as it allows healthcare professionals to identify and address pain promptly, leading to improved outcomes and quality of life. One of the key reasons for the importance of early pain assessment is the potential impact of untreated or undertreated pain on a child's overall well-being. Pain can have significant physical, emotional, and psychological consequences for pediatric patients. It can interfere with their ability to engage in daily activities, disrupt sleep patterns, and negatively affect their mood and behavior. Untreated pain can also lead to increased stress, anxiety, and fear, which can further exacerbate the pain experience. By assessing pain early, healthcare professionals can intervene promptly to alleviate pain and prevent these negative consequences [24].

Similarly, in pediatric patients undergoing painful procedures, such as surgery or medical interventions, early pain assessment enables healthcare professionals to provide appropriate analgesia and minimize the potential long-term effects of untreated pain.

Children's pain experiences can vary based on factors such as their developmental stage, individual pain tolerance, and response to treatment. Regular pain assessments allow healthcare professionals to monitor the effectiveness of pain management interventions and make necessary modifications to ensure optimal pain control. This ongoing assessment also provides an opportunity to address any new or emerging pain issues promptly, preventing unnecessary suffering and improving the child's overall well-being. Furthermore, ongoing pain assessment in pediatric patients promotes a patient-centered approach to care. It allows healthcare professionals to actively involve children and their families in the pain management process, ensuring that their preferences, concerns, and goals are considered [30, 31]. This collaborative approach not only enhances the child's experience but also improves treatment adherence and overall satisfaction with care.

## 7. Neurobiological Basis of Pain Perception in Children

Pain perception is a complex phenomenon that involves the integration of sensory, emotional, and cognitive processes. Understanding the neurobiological basis of pain perception in children is crucial for developing effective interventions and treatments for pediatric pain.

### 7.1. Role of Pain Memories

Pain memories play an integral role in the development and maintenance of chronic pain in children. Advances in neuroimaging techniques have allowed researchers to investigate the neurobiological underpinnings of pain memories in children. Noel et al. conducted a review of the literature on children's memories of pain and highlighted the potential role of pain memories in the development and maintenance of chronic pain [32]. They emphasized the need for interdisciplinary research in this area to further understand the neurobiology of pain memories in children.

### 7.2. Circadian Rhythmicity of Pain Sensitivity

The rhythmicity of pain sensitivity in children has been a topic of interest in recent research. Daguet et al. investigated the circadian rhythmicity of pain sensitivity in humans and found that pain sensitivity follows a sinusoidal pattern, with a maximum during middle sensitivity and a linear increase over prolonged wakefulness [33]. These findings highlight the importance of considering the time of day in pain perception research and clinical practice.

### 7.3. Sex Differences in Pain Perception

Sex differences contribute to the individual variability in pain perception and its underlying neural mechanisms. Kim et al. studied network-level brain dynamics associated with pain sensitivity and pain interference in healthy people and discovered that pain sensitivity and pain interference were linked to within- and between-network functional coupling in the brain, with sex differences in the dynamics of these networks observed [34]. Understanding sex differences in pain perception can help tailor pain management strategies for children based on their specific needs.

### 7.4. Brain Structures and Functions

Neuroimaging studies have provided insights into the neurobiological basis of pain perception in children. Kaplan et al. examined the neurobiological antecedents of multisite pain in children and found altered brain structure and function in children who subsequently developed multisite pain. They observed increased neural activity in the superior parietal/primary somatosensory and motor cortices and decreased activity in the medial prefrontal cortex in children with multisite pain [35]. These findings suggest a neural vulnerability to developing chronic pain in children. The insular cortex, a brain region involved in pain processing, has also been implicated in individual pain thresholds. Neumann et al. conducted a study on the network properties and regional brain morphology of the insular cortex and found that both regional gray matter volume and local connectivity correlated with individual pain thresholds [36]. This highlights the importance of the insular cortex in modulating pain perception in children.

## 8. Acute and Chronic Pain Consequences on Physical Recovery

Pain is a common experience in children, and it can have significant consequences for their physical recovery. Acute pain, which is short-term and typically associated with injury or illness, can disrupt a child's ability to engage in physical activities and impede their overall recovery process. On the other hand, chronic pain, which persists for an extended period, can have long-lasting effects on physical functioning and quality of life in children [37].

Acute pain in children can result from various causes, such as surgical procedures, injuries, or medical conditions. The experience of acute pain can have immediate consequences for a child's physical recovery. For example, after surgical procedures, children may experience pain that limits their mobility and hinders their ability to engage in physical activities necessary for recovery [38, 39]. Pain intensity has been shown to impact recovery, with higher pain levels associated with slower physical recovery. Inadequate pain management during the acute phase can prolong the recovery process and delay the return to normal physical functioning [40]. Furthermore, acute pain can also affect a child's mood and emotional well-being, which in turn can impact their physical recovery. Research has shown that pain and mood are closely related, with changes in mood influencing pain perception and physical function. Children experiencing acute pain may exhibit decreased motivation, increased fatigue, and reduced engagement in physical activities, all of which can impede their recovery [39, 41]. Therefore, addressing both pain and mood during the acute phase is crucial for optimizing physical recovery in children.

Chronic pain in children refers to persistent or recurrent pain that lasts for more than three months. It can result from various conditions, such as musculoskeletal disorders, neuropathic pain, or chronic illnesses. The consequences of chronic pain on physical recovery in children are more complex and multifaceted compared to acute pain. Children with chronic pain often experience limitations in physical functioning and reduced participation in daily activities [42]. The persistent nature of chronic pain can lead to deconditioning, muscle weakness, and decreased range of motion, which further exacerbate physical impairments. As a result, children with chronic pain may have difficulties performing tasks that require physical exertion, such as sports, exercise, or even routine activities like walking or standing for prolonged periods [43]. The impact of chronic pain on physical recovery extends beyond the physical realm. It can also affect a child's psychological well-being, social interactions, and overall quality of life. Parents of children with chronic pain have reported significant emotional distress and lifestyle disruptions due to their child's condition [42]. The burden of managing their child's pain and the associated challenges can further impact the parents' own quality of life, which in turn can influence the child's recovery process [44]. The consequences of chronic pain on physical recovery in children are not limited to the immediate effects. Research suggests that chronic pain in childhood can have long-term implications for physical health and functioning in adulthood. The experience of chronic pain during childhood may shape pain memories and pain-related behaviors, potentially leading to the development of persistent pain conditions later in life [32]. Therefore, addressing chronic pain in children is crucial not only for their immediate physical recovery but also for preventing long-term consequences.

## 9. Psychological Impact of Pain on Pediatric Burn Patients

The psychological impact of pain on pediatric burn patients is a significant concern that can have long-lasting effects on their well-being and recovery. Burn injuries in children can result in significant physical pain, but they also have profound psychological implications. The experience of pain can lead to various psychological responses, including anxiety, fear, depression, and post-traumatic stress symptoms. Children with burn injuries often face challenges in coping with their pain and adjusting to the changes in their physical appearance and daily routines. The psychosocial impact of burns on pediatric patients can be extensive, affecting their self-esteem, body image, social interactions, and overall quality of life. The psychological consequences of burns can persist long after the physical wounds have healed, leading to ongoing emotional distress and impaired functioning. Studies have shown that pediatric burn patients are at a higher risk of developing post-traumatic stress symptoms or post-traumatic stress disorder (PTSD). The traumatic nature of burn injuries, the pain associated with treatment procedures, and the potential for disfigurement can contribute to the development of PTSD symptoms. These symptoms may include intrusive thoughts, nightmares, avoidance behaviors, and hyperarousal [9].

Scarring caused by burn injuries can have an influence on the self-esteem of pediatric survivors. It is worth noting, however, that female burn survivors reported higher body esteem on average than females in the comparison group. Body esteem was shown to have no link with demographic characteristics, but it did have a tiny but significant relationship with scar severity, perceived stigmatization, and social comfort [45].

Psychological interventions play a crucial role in addressing the psychological impact of pain on pediatric burn patients. Evidence-based psychological interventions, such as cognitive-behavioral therapy (CBT), have shown promise in reducing pain-related distress and improving coping skills in children with chronic pain conditions. CBT focuses on identifying and challenging negative thoughts and beliefs about pain, teaching relaxation techniques, and promoting adaptive coping strategies [46, 47]. Virtual reality (VR) has also emerged as a potential tool for managing pain and anxiety in pediatric burn patients.

## 10. Pharmacological Management of Pain in Pediatric Burns

Pharmacological interventions play a crucial role in alleviating pain and improving the overall comfort of pediatric burn patients.

### 10.1. Opioid Analgesics

Opioid analgesics are commonly used in the management of acute pain in pediatric burn patients. These medications, such as morphine, fentanyl, hydrocodone, or codeine, provide potent pain relief by acting on opioid receptors in the central nervous system. Opioids are typically administered intravenously or through patient-controlled analgesia (PCA) pumps to ensure adequate pain control. However, the use of opioids in pediatric burn patients requires careful monitoring due to the potential for side effects such as respiratory depression and sedation [48]. Therefore, healthcare providers must balance the need for pain relief with the potential risks associated with opioid use.

### 10.2. Nonsteroidal Anti-Inflammatory Drugs (NSAIDs)

NSAIDs, such as ibuprofen or ketoprofen, are commonly used as adjuncts to opioid analgesics in the management of pain in pediatric burn patients. These medications provide analgesic and anti-inflammatory effects by inhibiting the production of prostaglandins, which are involved in the inflammatory response. NSAIDs can help reduce pain intensity and inflammation, thereby improving the overall comfort of pediatric burn patients. However, it is important to consider the potential side effects of NSAIDs, such as gastrointestinal bleeding or renal impairment, especially in patients with pre-existing conditions or prolonged use [15].

### 10.3. Local Anesthetics

Local anesthetics, such as lidocaine or bupivacaine, can be used to provide targeted pain relief in pediatric burn patients. These medications can be administered topically or through regional nerve blocks to numb the affected area and reduce the pain sensation. Local anesthetics are particularly useful during wound dressing changes or minor procedures, as they can provide immediate pain relief. However, the duration of action of local anesthetics is limited, and repeated administration may be necessary to maintain pain control [49].

### 10.4. Adjuvant Medications

Adjuvant medications, such as antidepressants or anticonvulsants, can be used in the management of neuropathic pain in pediatric burn patients. Neuropathic pain, which can occur as a result of nerve damage from a burn injury, can be challenging to treat with traditional analgesics alone. Medications such as gabapentin or amitriptyline can help modulate the abnormal pain signals associated with neuropathic pain and provide additional pain relief [15]. Some centers prefer to use nasal Dexmedetomidine combined with rectal ketamine for sedation and analgesia during burn wound care procedures such as dressing changes, which seem to minimize the need for orotracheal intubation [50]. However, the use of adjuvant medications in pediatric burn patients requires careful consideration of potential side effects and individual patient factors.

## 11. Nonpharmacological Approaches for Pain Management

Nonpharmacological approaches can also play a significant role in the management of pain in pediatric burn patients. Techniques such as distraction, relaxation exercises, or virtual reality (VR) have shown promise in reducing pain perception and improving the overall comfort of pediatric patients [51–53].

Virtual reality has emerged as a potential tool for managing pain and anxiety in pediatric burn patients. Studies have shown that VR can effectively distract children during painful procedures, reducing pain perception and anxiety. The immersive and interactive nature of VR provides a novel and engaging experience that can help children shift their attention away from the pain and discomfort associated with burn treatments. In addition to psychological interventions, effective pain management strategies are essential for addressing the psychological impact of pain on pediatric burn patients. Multimodal approaches that combine pharmacological interventions with nonpharmacological techniques, such as distraction, relaxation, and guided imagery, can help alleviate pain and reduce psychological distress [51, 52, 54].

Alongside this fairly new and somewhat expensive procedure of using VR as an adjunct for pain relief, the more common phone or tablet seems to reduce the pain and anxiety levels of patients undergoing hydrotherapy sessions under the supervision of a child life specialist [55].

## 12. Evidence-Based Guidelines for Multimodal Pain Management

Multimodal pain management, which involves the use of multiple interventions or medications targeting different aspects of the pain pathway, has gained recognition as an effective approach for managing pain in both children and adults. However, it is important to consider the specific needs and considerations of pediatric patients when developing evidence-based guidelines for multimodal pain management in this population.

### 12.1. Knowledge and Attitudes of Pediatric Clinicians

Research has shown that the knowledge and attitudes of pediatric clinicians play a crucial role in providing adequate pain management for children. Studies have highlighted the need for further education and training regarding pediatric pain management among pediatric clinicians. Knowledge deficits and misconceptions about pediatric pain can impact the ability of clinicians to provide effective pain relief [56]. Therefore, evidence-based guidelines for multimodal pain management in children should address the specific educational needs of pediatric clinicians to ensure optimal pain management outcomes.

### 12.2. Quality of Guidelines

The quality of clinical practice guidelines for pain management in children has been evaluated in several studies. A systematic review by Lee et al. assessed the quality of existing guidelines for acute procedural pain in children and found that the majority of guidelines were of average quality. The authors emphasized the need for more transparency and comprehensive reporting in the guideline development process [57]. This highlights the importance of developing evidence-based guidelines that are rigorous, transparent, and based on the best available evidence.

### 12.3. Barriers to Pediatric Pain Management

Several studies have identified barriers to pediatric pain management, which should be considered when developing evidence-based guidelines. Li et al. identified barriers such as misconceptions about pediatric pain, a lack of professional knowledge and confidence in pain assessment and relief interventions, and knowledge gaps between pediatricians and nurses. These barriers highlight the need for multidisciplinary education and training to address the specific challenges of pediatric pain management [58]. Evidence-based guidelines should address these barriers and provide recommendations for overcoming them to ensure optimal pain management in children.

### 12.4. Comparison with Adult Guidelines

While there is overlap between the principles of multimodal pain management in children and adults, there are also important differences. Pediatric patients may require age-appropriate dosing and formulations of medications, as well as specific considerations for pain assessment and communication. In addition, the use of certain interventions or medications may be limited in pediatric populations due to safety concerns or a lack of evidence. Therefore, evidence-based guidelines for multimodal pain management in children should consider these unique factors and provide specific recommendations tailored to the pediatric population.

## 13. Addressing Caregiver Distress Related to the Child's Pain

Children's pain can have a significant emotional and psychological effect on their caregivers. Research has shown that caregivers of children with chronic pain or conditions such as dental caries or other chronic illnesses report higher levels of distress. The constant worry and concern for the child's well-being, the challenges of managing the child's pain, and the disruption of daily routines can contribute to caregiver distress [59].

Caregiver distress related to the child's pain can significantly impact their own quality of life. Studies have shown that caregiver distress is associated with negative implications for their mental health, physical health, and overall well-being. Caregivers may experience increased stress, anxiety, depression, and decreased social support. The distress can also affect their ability to provide optimal care for the child, leading to a cycle of increased distress and compromised caregiving [60, 61].

Recognizing and addressing caregiver distress related to the child's pain is crucial for providing comprehensive care. Healthcare professionals should consider the following strategies.

### 13.1. Education and Support

Providing caregivers with education and support regarding the child's pain condition, pain management strategies, and coping techniques can help alleviate distress. This may include information about pain assessment, medication administration, and nonpharmacological interventions [59].

### 13.2. Communication and Empathy

Healthcare professionals should maintain open and empathetic communication with caregivers, acknowledging their concerns and providing reassurance. Regular check-ins and opportunities for caregivers to express their feelings and ask questions can help alleviate distress [59].

### 13.3. Referral to Support Services

Referring caregivers to support services, such as counseling, support groups, or respite care, can provide them with additional resources and assistance in managing their distress. These services can offer a safe space for caregivers to share their experiences and receive emotional support [60].

### 13.4. Collaborative Care

Adopting a collaborative approach to care, involving both the child and the caregiver, can help address the needs of both parties. This may involve joint decision-making, shared goal-setting, and involving caregivers in the child's pain management plan [60].

## 14. Advances in Pain Research and Technology

Pediatric pain is a complex issue that requires ongoing research and advancements in technology to improve pain assessment and management in children. Over the years, there have been significant advances in pediatric pain research and technology, leading to a better understanding of pain mechanisms and the development of innovative approaches to assess and treat pain in children.

### 14.1. Sleep and Pediatric Pain

One area of advancement in pediatric pain research is the recognition of the relationship between sleep and pain in children. Research by Valrie et al. highlights the importance of sleep in pediatric pain populations. The systematic review found evidence for a pain-sleep relationship in children with persistent pain. Poor sleep quality and disrupted sleep patterns were associated with increased pain severity and functional impairment in children with persistent pain. This understanding has led to the inclusion of routine sleep screenings and the use of standardized measures of sleep in pediatric pain management [62]. By addressing sleep disturbances, healthcare providers can improve pain management outcomes and enhance the overall well-being of children with pain.

### 14.2. Technology in Pain Assessment

Advancements in technology have also played a significant role in pediatric pain assessment. The use of innovative tools and devices has improved the accuracy and objectivity of pain assessment in children. For example, the use of facial expression recognition software and wearable sensors has allowed for real-time monitoring and analysis of pain-related behaviors and physiological responses in children [63, 64]. These technological advancements provide valuable insights into the child's pain experience, allowing healthcare providers to tailor pain management strategies more effectively.

Distraction techniques, such as music therapy, guided imagery, or play therapy, can also divert a child's attention away from pain and promote relaxation. These nonpharmacological approaches offer safe and engaging alternatives to traditional pain management techniques, enhancing the overall experience for children undergoing painful procedures [31, 55].

Advancements in pharmacological approaches have also improved pediatric pain management. The development of age-appropriate formulations and dosing guidelines for analgesic medications has facilitated safe and effective pain relief in children [65]. In addition, the use of adjuvant medications, such as antidepressants or anticonvulsants, has shown promise in managing neuropathic pain in pediatric populations [58]. These pharmacological advancements provide healthcare providers with a wider range of options to tailor pain management strategies to the specific needs of each child.

## 15. Conclusion

The treatment of pediatric burn injuries is a complex process that requires intensive therapeutic and surgical interventions. Currently, the standard practice involves donor site harvest and autografting, but alternative treatments are urgently needed for both adult and pediatric populations. The management of burn wounds involves preventing sepsis and other complications that can further delay wound closure.

Pediatric burns have significant implications for both the physical and psychological well-being of affected children. The impact of burn injuries on skeletal muscle protein synthesis, cardiovascular function, body esteem, and treatment strategies are areas of ongoing research. Understanding these aspects is crucial for improving the long-term outcomes and quality of life for pediatric burn survivors.

Comprehensive pain management guidelines for pediatric burn patients should take into account factors that influence pain levels, such as the severity of the burn injury and the individual characteristics of the patient. Overall, pain management in pediatric burns requires a multidisciplinary approach that combines pharmacologic and nonpharmacologic interventions. The use of VR games, analgesic medications, and careful management of donor site wounds are important strategies for providing effective pain relief for pediatric burn patients. Further research and advancements in pain management techniques are needed to improve the outcomes and quality of life for these young patients.
